# Atypical hemolytic uremic syndrome in a patient with HIV treated with eculizumab: A case report

**DOI:** 10.1016/j.idcr.2023.e01692

**Published:** 2023-01-14

**Authors:** Danilo E.Trujillo González, Dagoberto Duarte Misol, Nicolás Ariza Ordoñez, Fabian Andres Salgado Zamora, Henry A. Millan Prada, Eduardo Zuñiga R, Alejandra Molano Triviño

**Affiliations:** a Universidad del Rosario, Bogotá, Colombia; b Universidad del Norte, Barranquilla, Colombia; c La Cardio, Bogotá, Colombia

**Keywords:** Thrombotic microangiopathy, Atypical hemolytic uremic syndrome, Human immunodeficiency virus, Eculizumab, Plasma therapeutic exchange, Acute kidney injury

## Abstract

Thrombotic microangiopathy defines a group of pathologies characterized by microvascular dysfunction with the concurrence of microangiopathic hemolytic anemia, thrombocytopenia, and organ damage. It represents the most frequent microvascular manifestation of human immunodeficiency virus (HIV) infection. We report the case of a man in the seventh decade of life with a recent diagnosis of infection by HIV, who develops hemolytic uremic syndrome, requiring continuous renal replacement therapy and plasma replacement therapy, without response, ADAMTS13 with preserved activity, ruling out other etiologies (infectious, metabolic, and genetic) with successful response to eculizumab.

## Introduction

Thrombotic microangiopathy (TMA) defines a heterogeneous group of pathologies with microvascular dysfunction characterized by the concomitance of microangiopathic hemolytic anemia, thrombocytopenia, and organ damage. It is the most frequent microvascular manifestation of human immunodeficiency virus (HIV) infection, especially in advanced stages with opportunistic infections, high viral loads, and low CD4 lymphocyte counts. The spectrum of presentation of this relationship is varied, with thrombotic thrombocytopenic purpura being the most frequent form [1].

We present the case of a 60-year-old patient with a recent diagnosis of HIV infection who was diagnosed with secondary TMA, with severe renal involvement related to hemolytic uremic syndrome (HUS), and a favorable clinical response to anti-complement therapy with eculizumab. This case highlights the unusual presentation and etiology of TMA, demonstrating the importance of recognizing therapeutic options in primary and secondary settings.

## Case

A 64-year-old male patient with recent diagnosis of human immunodeficiency virus infection (unstaged and without retroviral management). He was admitted to the emergency department complaining of 15 days with intermittent fever in up to 39ºC associated with nocturnal diaphoresis, asthenia, adynamic, and progressive symmetrical edema of the lower limbs. Also, he referred chronic diarrhea that resolved before admission. He reported smoking 20 cigarettes per day for 20 years (20 pack-year smoking history). Furthermore, he received 2 months earlier the first dose SARS-CoV 2 vaccine (Sinovac) and reported an unintentional loss of 4 kg in the last 5 months. He denies any other relevant personal or family history. Physical examination revealed tachycardia, tachypnea, hypotension, without hypoxemia, severe dehydration, pallor, normal cardiopulmonary auscultation, absence of abdominal pain with grade III edema in lower limbs without any neurological focalization.

**Fig. 1:**
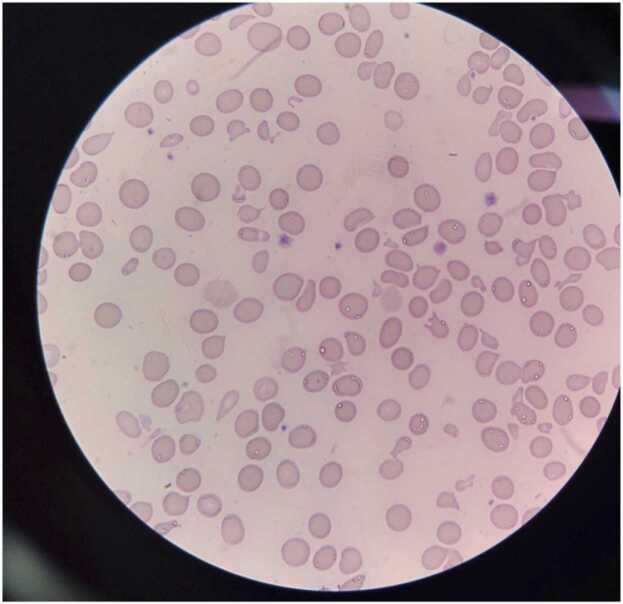
Peripheral blood smear. Mild anisocytosis, anisochromia, and poikilocytosis with schistocytes.

Sepsis was considered and empirical antibiotic coverage was started with Metronidazole and Ampicillin/Sulbactam (with subsequent adjustment accord to the renal function). Cultures of blood, urine, lower respiratory tract, and stool were taken before antimicrobial therapy was administrated without any isolation, as well as multiple detection panels of the gastrointestinal and lower respiratory tract. Due to lack of response to crystalloids, he was transferred to the Intensive Care Unit (ICU) for high-dose vasopressor infusion. The patient developed refractory shock with subsequent multisystem failure and required ventilatory support. Blood tests reports (Table 1) documented severe hypochromic non-regenerative microcytic anemia (corrected reticulocytes of 1.4 %), severe thrombocytopenia, and progressive increase in creatinine with anuria and volume overload requiring venovenous hemodiafiltration. Complementary studies revealed elevated lactate dehydrogenase (LDH), negative direct Coombs and iron deficiency anemia. Peripheral blood smear showed schistocytes (Fig. 1). A bone marrow biopsy did not reveal any evidence for neoplastic or infectious processes. HIV viral load was 622.148 copies/mL – Log 5.79 and CD4^+^ 42 cells/µL. Due to quantitative serum cytomegalovirus (CMV) PCR level increased from 35 copies/mL - 1.54 log to 104 copies/mL – 2.04 log, ganciclovir was started.

**Table 1:** Blood tests.

	Admission	48 h post-admission	At the beginning of PET	During PET	48 h PET suspension	At the beginning of eculizumab	At the fourth dose of eculizumab
Hemoglobin (gr/dl)	5.1	9.4	9.9	7.1	5.09	6.93	8.91
Leucocytes (10^3cel/u)	9.24	10.6	4.48	1.34	2.6	3.36	2.37
Platelets (10^3cel/u)	77.3	45.7	7.4	36.4	6.3	5.12	88.2
Creatinine (mg/dl)	1.1	0.8	2.7	2.3	2.6	2.2	1.0
Blood urea nitrogen (mg/dl)	29	28	116	63	93	105	30
ALT (U/l)	89	37	23	52	50	57	-
AST (U/l)	107	62	325	89	59	76	-
Alkaline phosphatase (U/l)	303	201	261	123	126	139	-
GGT (U/l)	41	-	65	33	-	-	-
Albumin g/dl	1.9	1.8	2.4	-	-	-	-
INR	1.3	1.33	1.19	1.27	1.39	1.1	1.18
Bilirubin (mg/dl)	TB:0.7; DB:0.4; IB:0.3	TB:1.0; DB:0.6; IB:0.4	TB:31.2; DB:20.6; IB:10.6	TB:5.2; DB:3.4; IB:1.8	TB:7.4; DB:5.5; IB:1.9	TB:14.3; DB:10.8; IB:3.5	TB:4.4; DB:3.5; IB:0.9
Lactate dehydrogenase (U/l)	299	-	-	305	-	-	215

ALT: Alanine-amino transferase; AST: Aspartate aminotransferase; TB: Total bilirubin, DB: Direct bilirubin, IB: Indirect bilirubin; GGT: Gammaglutamyl Transferase; INR: International Normalized Ratio; PET: Plasma exchange therapy

The diagnosis considered at this time was secondary thrombotic microangiopathy/atypical hemolytic uremic syndrome. Due to persistent anemia and thrombocytopenia with multiple transfusion requirements during his stay at the ICU, plasma exchange therapy (PET) was started (negative report of Shiga-Toxin on a film array panel and a PLASMIC score of 6 points). We observed an improvement in platelet count (although not LDH) after 13 sessions of PET, but after anemia and severe thrombocytopenia recurred, 6 more sessions of PET were administrated. At this point the patient completed 10 days of Metronidazole + Ampicillin/Sulbactam; 14 days of Amphotericin B (empirical, with the suspect of invasive fungal disease), and current treatment with ganciclovir (10 days so far). In the search for other causes of anemia in a critically ill patient, esophagogastroduodenoscopy was performed, and biopsy of polyps found in the gastric antrum was compatible with Kaposi's sarcoma. Bone marrow cultures, biopsy, and flow cytometry revealed preserved hematopoiesis of all three lines with no pathological findings.

A cholestatic pattern was discovered so an MR cholangiography and pancreatography was performed to rule out obstruction of the bile duct and infiltrative involvement, observing findings suggestive of hepatic iron overload due to possible secondary hemosiderosis. Anti-smooth muscle antibodies, anti-mitochondrial membrane antibodies, antinuclear antibodies, and hepatotropic viruses were performed with negative results. The percentage of transferrin saturation was less than 45 % and a liver biopsy was performed in which abundant iron deposits are observed in the cells of the reticuloendothelial system in addition to findings compatible with acute lobular hepatitis with cholestasis. Since we didn’t found evidence of cirrhosis, cholangiopathy, intrinsic hepatic infectious processes, or autoimmunity, hemosiderosis was attributed to multiple blood transfusions.

**Fig. 2:**
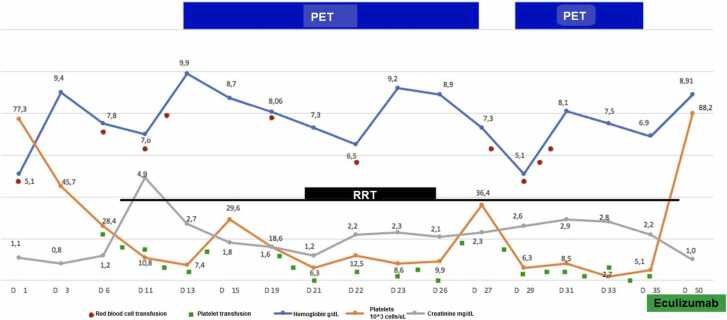
Trend of blood tests and interventions. PET: Plasma exchange therapy, RRT: Renal replacement therapy, D: Day.

In consensus led by the infectious disease services group, antiretroviral therapy was initiated (Abacavir 300 mg every 12 h + Lamivudine 150 mg every 12 h + Dolutegravir 50 mg every day) without any clinical improvement after 14 days. Considering the requirement for PET, the ADAMTS13 of 34.8 %, and the futility of the treatment so far used, we initiated eculizumab 900 mg (4 sequential doses, with the previous immunization against *pneumococcus* and *meningococcus*). We observed improvement of the hematological profile, increase in urinary volume, and decrease the creatinine value (Fig. 2). This treatment allowed us to wean the extracorporeal support –renal and ventilatory- along with the vasoactive medication.

In conclusion, our patient presented with clinical and laboratory evidence of TMA in the context of HIV infection stage C3/AIDS, associated with multi-organ dysfunction, refractory shock, and unsatisfactory evolution, despite ruling out other etiologies of shock (cortisol in normal ranges), who received multiple broad-spectrum antimicrobial regimens without clinical response. We consider this patient’s clinical course is compatible with Atypical Hemolytic Uremic Syndrome secondary to HIV infection. A report of genetic sequencing by Next Generation Sequencing (NGS) perform in serum was negative for pathogenic, probably pathogenic, or variants of uncertain clinical significance identified in the complement coding regions. The patient was discharged with recommendation to continue eculizumab on an outpatient basis and follow-ups by the hematology and infectious disease services. At the publication date, the patient attended to hematology’s office one month after to discharge without requiring dialysis, and with minor thrombocytopenia and anemia.

## Discussion

Thrombotic microangiopathies (TMA) comprise a set of clinical syndromes characterized by the presence of microangiopathic hemolytic anemia, thrombocytopenia, and organ damage. These characteristics are secondary to vascular damage manifested as capillary and arteriolar thrombosis, with characteristic changes at the endothelium and the vessel wall. Historically they have been classified as primary or secondary according to their etiology [1]. Primary TMAs include thrombotic thrombocytopenic purpura (TTP), complement-mediated TMA (also called Atypical Hemolytic Uremic Syndrome), and rare hereditary pathologies related to vitamin B12 metabolism or proteins involved in hemostasis [2]. Secondary TMA is usually associated with systemic diseases including hypertensive disease related to pregnancy (preeclampsia and HELLP syndrome), infections, medications, neoplasms, autoimmune diseases, and as a complication of solid organ or hematopoietic stem cell transplantation. Clinical manifestations are several depending on the underlying disease, including acute kidney injury (requiring renal replacement therapy in a significant proportion of patients), neurological symptoms (including altered consciousness and seizures), myocardial disease, pulmonary compromise, digital gangrene, and eye involvement.

Bayer et al. published a series of 564 patients diagnosed with TMA in four French hospitals. Primary entities were identified in only 6 % of cases, while in the remaining 94 % TMA was diagnosed secondary to infections (33 %), medications (26 %), malignancy (19 %), and autoimmune diseases (9 %) as the most frequent etiologies. TMAs associated with infections constitute a diverse entity with multiple pathophysiological mechanisms depending on the pathogen involved. They have been frequently related to bacterial (especially those caused by Shiga toxin-producing *Escherichia coli* and other gram-negative bacilli, *Staphylococcus*, *pneumococcus)*, viral (especially by Epstein-Barr virus, Cytomegalovirus, Hepatitis C, Influenza and HIV) and to a lesser extent, parasitic and fungal infections [3].

Based on the clinical history and relevant diagnostic tests, we concluded that our patient had TMA associated with HIV infection. Despite the initiation of antiretroviral therapy and plasma exchange, a clinical response with platelet count recovery was only obtained with the initiation of complement blockade therapy.

In the setting of HIV infection, a higher incidence of TMA has been documented compared to the general population, an association initially described in 1984, especially in advanced stages of the disease and in some cases as its initial presentation [4]. The exact mechanism of the pathogenesis of HIV-related TMA is speculative. However, several infection-related factors may be associated with endothelial damage or micro thrombosis (essential pathologic features of TMA). Among them, the direct cytopathic damage induced by HIV in the endothelium, as well as the opportunistic pathogens, the related malignant neoplasms and the drugs that are used as treatment [5]. Although so far the relationship between aSHU and vaccination against SARS-CoV2 is weak, there are cases in which TMA has been identified as a manifestation of structural-histological and functional renal involvement, although the association with glomerular pathology precipitated by immunization against the virus is more common [6], [7], [8], [9]. In this case, aSHU associated with vaccination used to prevent COVID-19 was not considered because of the temporality between the events.

The risk factors identified with TMA as a manifestation of endothelium damage in HIV include: high viral loads (median 89,500 copies/mL), low levels of CD4+ lymphocytes (median 58 cells per µL), and a higher incidence of concurrent infections at presentation, especially those caused by the complex of *Mycobacterium avium* and hepatitis C [10]. Additionally, a trend toward lower platelet counts compared to HIV-negative patients has been documented [11]. Considering the spectrum of clinical presentation, in a study that included 20 patients diagnosed with TMA and HIV infection, 70 % had severely reduced levels of ADAMTS13 activity consistent with a diagnosis of TTP, while 30 % had normal levels of ADAMTS13 activity concordant with the HUS spectrum [12].

The absence of neurological manifestations, the severe renal involvement, and the preserved activity of ADAMTS13 led us to consider the possibility of complement-mediated TMA secondary to HIV infection therefore the use of eculizumab was proposed, with dramatic clinical results. This humanized monoclonal antibody that binds to the C5 protein, preventing its passage to C5a and C5b, blocking complement-mediated endothelial damage has been used in the past on patients with secondary TMA to HIV, with variable clinical results and a high rate of recurrence [13], [14]. It is for this reason that indefinite treatment was considered in the patient described.

In summary, in patients with TMA associated with HIV presenting within the spectrum of HUS, combined therapy with eculizumab and initiation of ART may be beneficial. The decision to discontinue anti-complement therapy is not yet supported by clinical trials but may be considered in the context of resolution of TMA and complete virologic response to antiretroviral therapy.

## Ethical approval

The manuscript and informed consent were submitted to the ethics committee of the institution La Cardio - Fundación Cardioinfantil and approval was obtained to publish the case.

## Consent

Studies on patients or volunteers require ethics committee approval and fully informed written consent which should be documented in the paper.

## CRediT authorship contribution statement

Danilo E. Trujillo González: Writing, Data collection. Dagoberto Duarte Misol: Writing, Searching, Data collection. Nicolás Ariza Ordoñez: Writing, Data Collection. Fabian Andres Salgado Zamora: Writing, Others. Henry A. Millan Prada: Writing, Others. Eduardo Zuñiga R: Writing, Analysis of the case. Alejandra Molano Trivño: Writing, Analysis of the case.

## Declaration of interest

The authors expressly declare that there is no conflict of interest relevant to the preparation and construction of this document. The patient gave his informed consent for purely scientific purposes. The manuscript and informed consent were submitted to the ethics committee of the institution La Cardio - Fundación Cardioinfantil and approval was obtained to publish the case.
